# Supplementary material for: Integration of affective cues in context-rich and dynamic scenes varies across individuals
Source: Nat Commun. 2025 Dec 16;17:786. doi: 10.1038/s41467-025-67466-1 (PMC12824156; doi:10.1038/s41467-025-67466-1)
Supplement: Supplementary file 1 — Supplementary Information [file 41467_2025_67466_MOESM1_ESM.pdf]

Supplementary information for

Integration of affective cues in context-rich and dynamic scenes varies across individuals

Jefferson Ortega[1], Yuki Murai[2], David Whitney[1,3,4]

[1] Department of Psychology, University of California, Berkeley, CA 94720, USA
[2]Center for Information and Neural Networks, National Institute of Information and Communications Technology, Osaka, Japan
[3]Helen Wills Neuroscience Institute, University of California, Berkeley, CA, USA
[4]Vision Science Group, University of California, Berkeley, CA, USA

* Corresponding author. Email: Jefferson_ortega@berkeley.edu

Individual differences in integration strategies

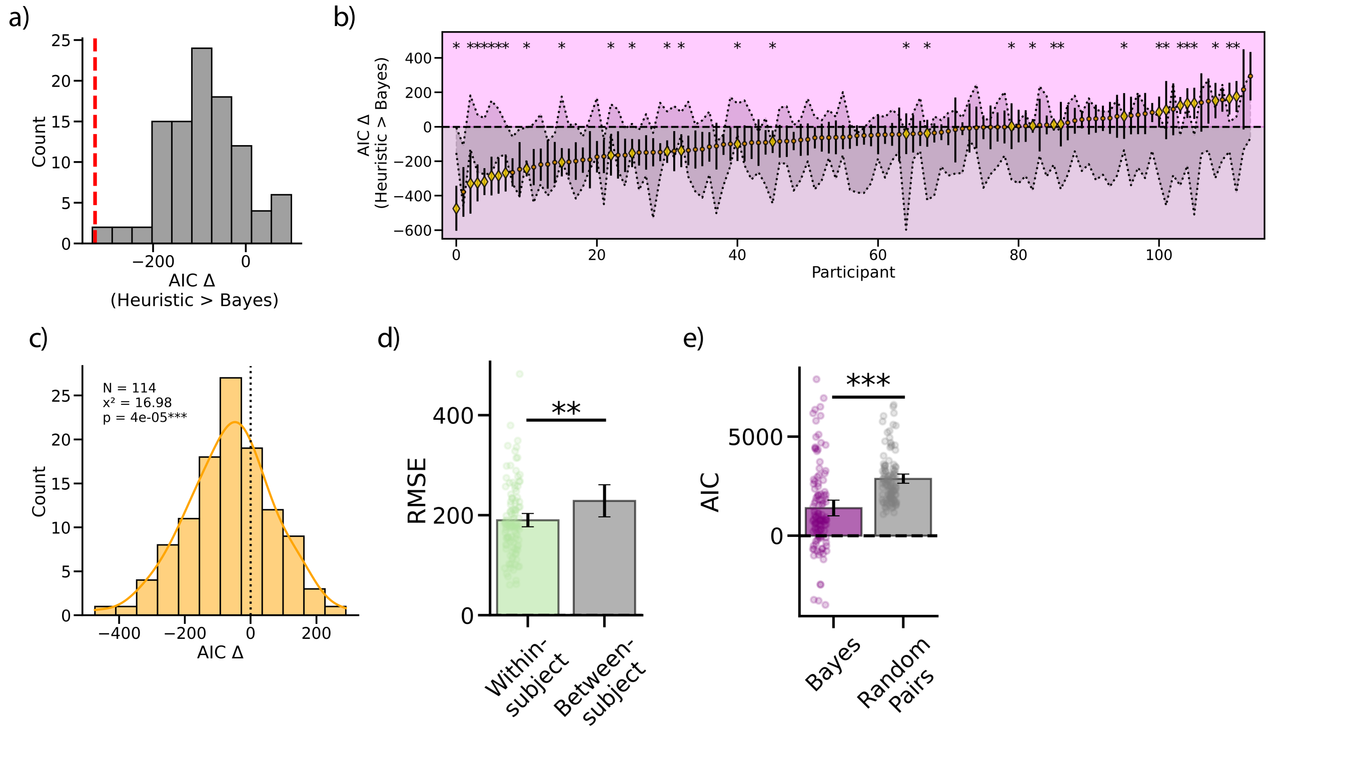

Figure S1 | Individual differences in integration strategies in Experiment 3 (N = 124). We investigated whether there were individual differences in integration strategies by plotting model performance between the Bayesian and Heuristic models for each individual participant. We first computed the differences between the Akaike information criterion (AIC) values of the Bayesian model and the Heuristic model for each participant. Positive values indicate that the participants data was best captured by the Heuristic model while negative values indicate the Bayesian model best captured their ratings. We then compared the AIC difference scores to a permuted null distribution. The permuted null was computed by shuffling observers' ratings such that a random observer's context and character ratings were integrated to predict each observers ground truth rating. This allows us to investigate whether there are significant idiosyncratic differences within observers, or if observers' data is interchangeable between observers. **(a)** shows an example of the AIC difference scores obtained for a single observer's permutation (gray distribution; 100 iterations) compared to their observed AIC difference score (vertical red dashed line). **(b)** shows the same analysis for all observers where the shaded region is the 95% confidence interval (CI) of the permuted null distribution. Observers who had significant AIC differences (those that were outside of the permuted 95% CI) are shown as a yellow diamond and have an asterisk (*) at the top of the figure. In total, there were 30 observers who had significant individual differences which is significantly more than expected by chance ($\chi2 = 101$, $p < 0.001$). **(c)** We also found that more individual observers were best modeled by the Bayesian model than the Heuristic model ($\chi2=16.98$, $p < 0.001$). **(d)** To further explore whether there were significant individual differences within observers, we performed a split-half analysis where we split each observer's AIC difference scores in half, randomly, and compared the average AIC difference score for each half for 10,000 iterations using RMSE (within-subject analysis). We compared this to a between-subject analysis where we randomly selected a pair of observers AIC differences scores and compared the average AIC difference score for 10,000 iterations using RMSE (between-subject analysis).

Individual data points are not shown for the between-subjects data as it was calculated using permutation. We find that AIC differences are more similar within-subjects than between-subjects, providing further evidence for individual differences among observers (p = 0.0072; permutation test). **(e)** Finally, we investigated how the performance of the Bayesian model differs if we were to shuffle context and character ratings across observers. We find that integration cues retrieved from a random pair of observers lead to worse model performance than when using each observers own ratings for character and context cues (t(113) = -12.72, p < 0.001). Overall, these findings suggest that different observers may use different integration strategies when combining affect information, highlighting the presence of idiosyncratic differences across observers. Error bars represent bootstrapped 95% confidence intervals derived from 5,000 iterations.

Model performance for valence and arousal dimensions

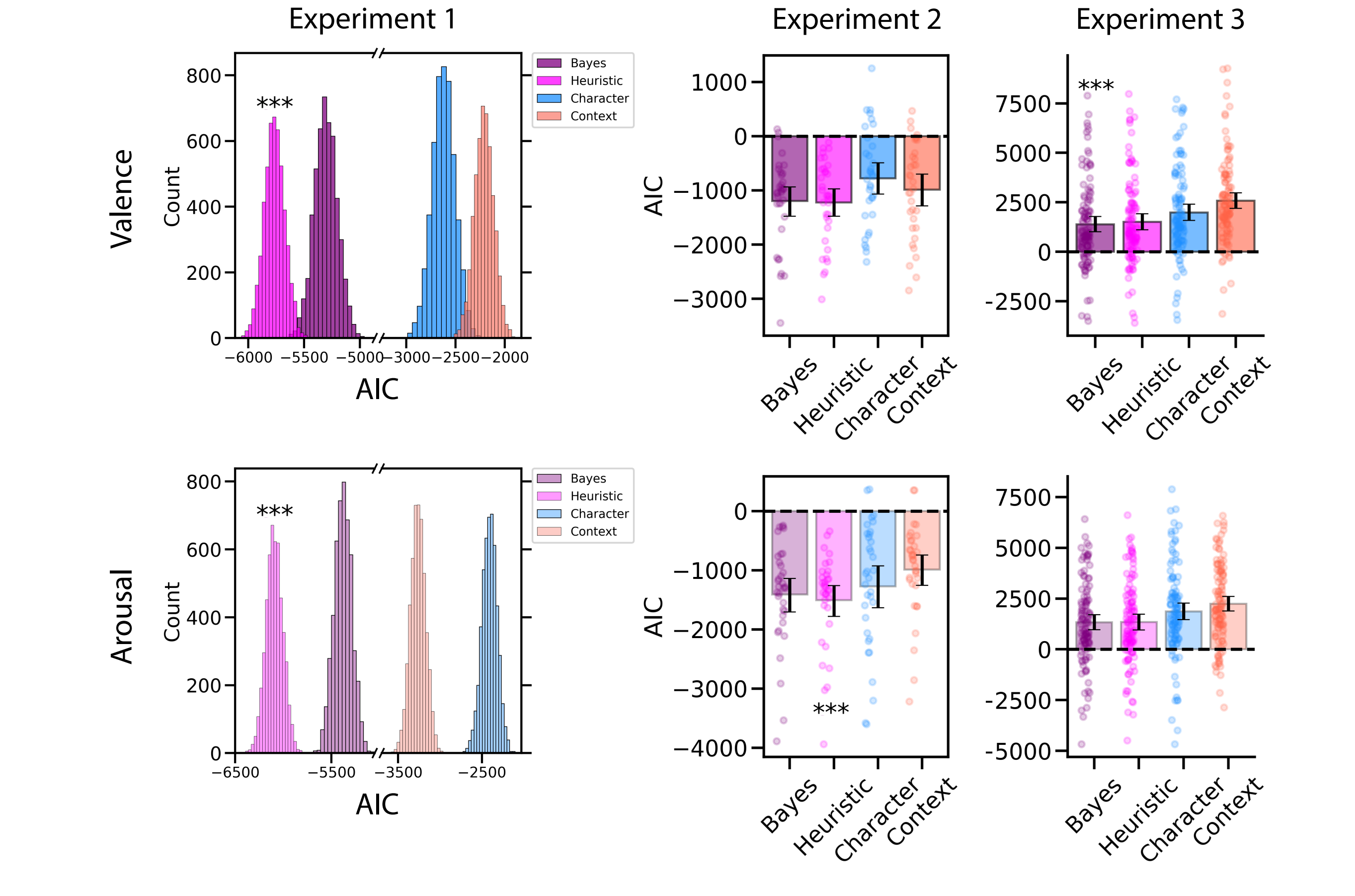

Figure S2 | Model performance for valence and arousal dimensions. We investigated model performance for both valence and arousal individually to explore whether there were differences in performance across affect dimensions. To compare model performance in Experiment 1 (N = 593), we bootstrapped parameter estimates and 95% confidence intervals. We find that the Heuristic model outperformed the Bayesian model in both valence and arousal dimensions in Experiment 1. In Experiment 2 (N = 227), we find no difference in performance between the Bayesian and Heuristic models in the valence dimension (t(67) = 0.52, p = 0.603) but we find a significant difference in performance for the arousal dimension (t(67) = 1.87, p = 0.07). Finally, we find a significant difference

in performance between the Bayesian and Heuristic models when predicting observers' ratings in valence ($t(113) = -6.77$, $p < 0.001$) but not in arousal ($t(113) = -0.44$, $p = 0.662$) dimensions in Experiment 3 (N = 124). Error bars represent bootstrapped 95% confidence intervals derived from 5,000 iterations.

Correlations between context and character ratings

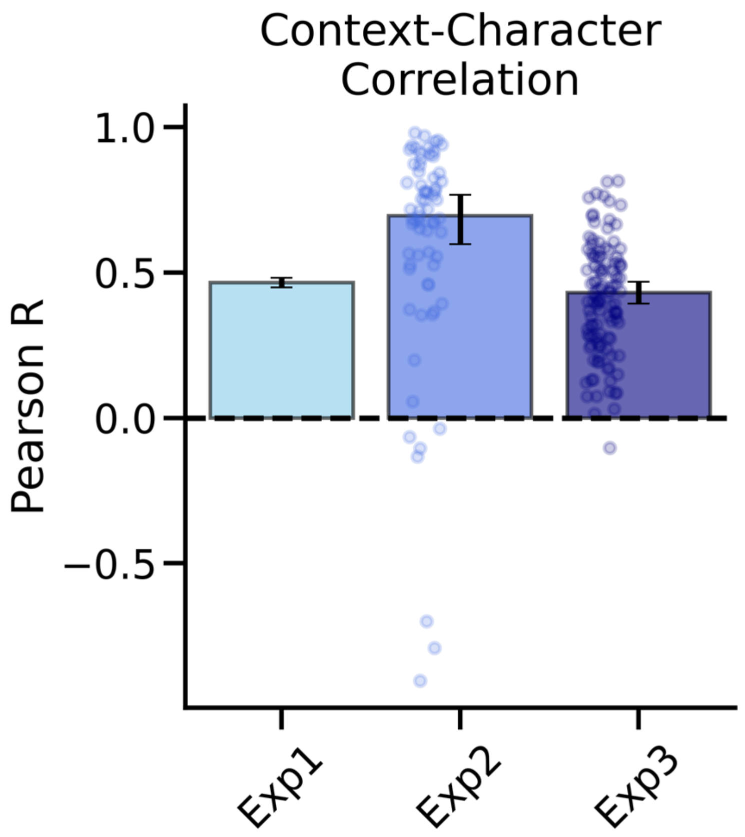

Figure S3 | Correlations between context and character ratings. Here we calculated the correlation between context ratings and character ratings across all experiments. Note that the information that observers see in both the context and character only conditions contain independent information. Experiment 2 (N = 227) had the highest context-character correlation (Mean: 0.691, 95% CI: [0.599, 0.766]) followed by Experiment 1 (Mean: 0.498, 95% CI: [0.478, 0.518] and then Experiment 3 (Mean: 0.430, 95% CI: [0.415, 0.446]). Error bars represent bootstrapped 95% confidence intervals derived from 5,000 iterations. Individual data is not shown for Experiment 1 as it was calculated from bootstrapping.

Non-parametric Bayesian integration using adaptive kernel density estimation

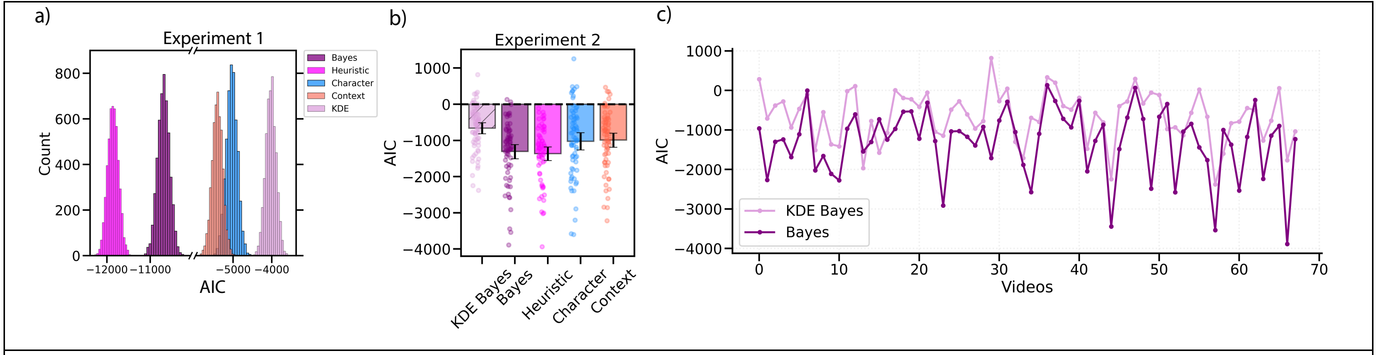

Figure S4 | Non-parametric Bayesian integration using adaptive kernel density estimation. In order to investigate whether affective cue integration may follow non- parametric Bayesian integration, we tested a model that used adaptive kernel density estimation (KDE) to fit distributions to the data. **(a)** We find that a model that uses adaptive KDE (AIC = -4026, 95% CI: [-4295,-3763]) Bayes performed much worse than the Bayesian integration model (AIC = -6669, 95% CI: [-6892, -6447]) and the Heuristic model (AIC = - 7590, 95% CI: [-7788, -7385]) in Experiment 1. **(b)** In Experiment 2 (N = 227), the adaptive KDE model (AIC: -660, 95% CI: [-814, -512]) again performed worse than the Bayesian (AIC: -1304, 95% CI: [-1509, -1110]; $t(67) = 7.89$, $p < 0.001$) and the Heuristic integration models (AIC: -1366, 95% CI: [-1556, -1184] $t(67) = 8.76$, $p < 0.001$). **(c)** Interestingly, in Experiment 2, we find that some videos (n=8) are best modeled by the adaptive KDE model suggesting that observers may try to estimate the true distribution of cues during certain conditions.

Individual video analysis

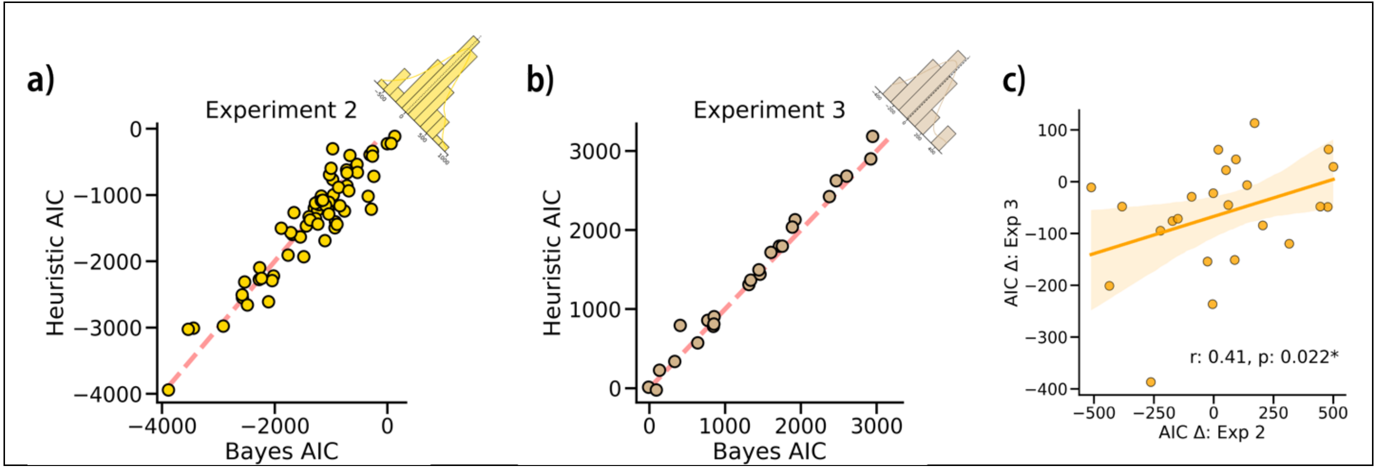

Figure S5 | Individual video analysis. We further investigated model performance for the Bayesian and Heuristic integration model by comparing performance across videos. (a) and (b) compare AIC values between the Bayes and Heuristic model for Experiment 2 (N = 227) and Experiment 3 (N = 124), respectively. The distribution in Experiment 2 were not significantly different from 0 ($x^2 = 2.12$, $p = 0.1456$) but the distribution for Experiment 3 was ($x^2 = 6$, $p = 0.0143$). These results provide further evidence suggesting that the Bayesian integration model outperformed the Heuristic model in Experiment 3 where affect ratings were modeled within observer. (c) shows the AIC difference between the Bayes and Heuristic models for videos that were included in both Experiment 2 and 3 (n = 12). Positive values indicate that the Heuristic model is better while negative values indicate the Bayesian model is better. We find a significant positive correlation between model performance differences for videos present in both Experiment 2 and Experiment 3 indicating that model performance may generalize across experiments for the same video (Spearman r = 0.41, p-value = 0.022; one-tailed test).

Model comparison using protected exceedance probabilities

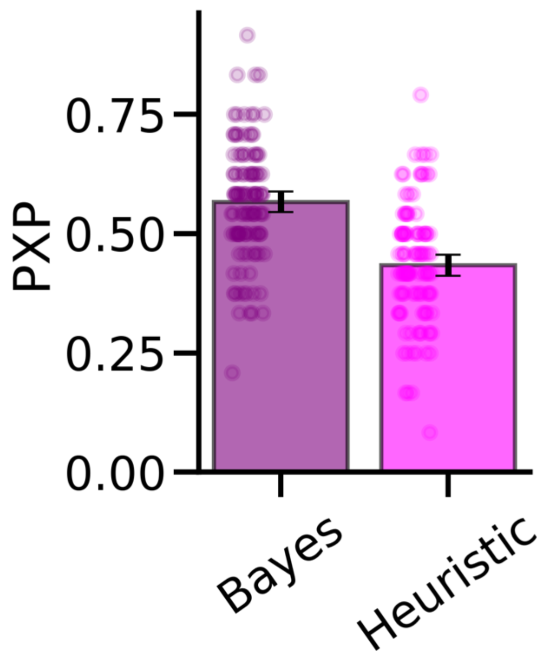

Figure S6 | Model comparison using protected probability exceedance. We further compared model performance in Experiment 3 (N = 124) by calculating protected exceedance probabilities (PXP) which is a measure of how likely any given model is compared with all other models in the comparison set[1,2]. We found that the Bayesian model (PXP: 0.567, 95% CI: [0.545, 0.589]) had higher PXP values than the Heuristic model (PXP: 0.433, 95% CI: [0.412, 0.455]), indicating that it better fit observers' ratings in Experiment 3 than the Heuristic model.

Variance predictions of ground truth ratings

Table S1. Model predictions of ground truth rating variance.

**Model**	Exp 1: Pearson r	Exp 1: RMSE	Exp 2: Pearson r	Exp 2: RMSE
**Bayes**	0.235	0.14	**0.483**	0.116
**Heuristic**	**0.26**	**0.047**	0.478	**0.053**

Note: Model with most accurate prediction in bold.
