# Supplementary material for: Integration of affective cues in context-rich and dynamic scenes varies across individuals
Source: Nat Commun. 2025 Dec 16;17:786. doi: 10.1038/s41467-025-67466-1 (PMC12824156; doi:10.1038/s41467-025-67466-1)
Supplement: Supplementary file 2 — Reporting Summary [file 41467_2025_67466_MOESM2_ESM.pdf]

nature portfolio

Corresponding author(s): Jefferson Ortega

Last updated by author(s): August 17, 2025

Reporting Summary

Nature Portfolio wishes to improve the reproducibility of the work that we publish. This form provides structure for consistency and transparency in reporting. For further information on Nature Portfolio policies, see our Editorial Policies and the Editorial Policy Checklist.

Statistics

For all statistical analyses, confirm that the following items are present in the figure legend, table legend, main text, or Methods section.

n/a	Confirmed	
☐	☒	The exact sample size (*n*) for each experimental group/condition, given as a discrete number and unit of measurement
☐	☒	A statement on whether measurements were taken from distinct samples or whether the same sample was measured repeatedly
☐	☒	The statistical test(s) used AND whether they are one- or two-sided<br>*Only common tests should be described solely by name; describe more complex techniques in the Methods section.*
☐	☒	A description of all covariates tested
☐	☒	A description of any assumptions or corrections, such as tests of normality and adjustment for multiple comparisons
☐	☒	A full description of the statistical parameters including central tendency (e.g. means) or other basic estimates (e.g. regression coefficient) AND variation (e.g. standard deviation) or associated estimates of uncertainty (e.g. confidence intervals)
☐	☒	For null hypothesis testing, the test statistic (e.g. *F*, *t*, *r*) with confidence intervals, effect sizes, degrees of freedom and *P* value noted<br>*Give P values as exact values whenever suitable.*
☐	☒	For Bayesian analysis, information on the choice of priors and Markov chain Monte Carlo settings
☒	☐	For hierarchical and complex designs, identification of the appropriate level for tests and full reporting of outcomes
☐	☒	Estimates of effect sizes (e.g. Cohen's *d*, Pearson's *r*), indicating how they were calculated

Our web collection on statistics for biologists contains articles on many of the points above.

Software and code

Policy information about availability of computer code

Data collection	Data for Experiment 3 was collected using PsyhoPy (v2022.1.1).
Data analysis	Analysis was conducted using Python (3.10.8) and Jupyter Notebook (5.2.0). Code used to analyze data for this project is publically avaialble on https:/ /osf.io/mrtv5/

For manuscripts utilizing custom algorithms or software that are central to the research but not yet described in published literature, software must be made available to editors and reviewers. We strongly encourage code deposition in a community repository (e.g. GitHub). See the Nature Portfolio guidelines for submitting code & software for further information.

Data

Policy information about availability of data

All manuscripts must include a data availability statement. This statement should provide the following information, where applicable:

- Accession codes, unique identifiers, or web links for publicly available datasets
- A description of any restrictions on data availability
- For clinical datasets or third party data, please ensure that the statement adheres to our policy

All data (deidentified) collected and analyzed in this study (Experiments 1-3) can be found on https://osf.io/mrtv5/

Research involving human participants, their data, or biological material

Policy information about studies with human participants or human data. See also policy information about sex, gender (identity/presentation), and sexual orientation and race, ethnicity and racism.

Reporting on sex and gender	Our analyses do not take into consideration sex or gender as it was not a variable of interest in our study. Participants in our study come from students at our university, specifically students who were, at the time, participating for course credit for a Psychology course. Sex and gender of the participants were recorded by having participants fill out a demographics questionnaire at the end of the experiment.
Reporting on race, ethnicity, or other socially relevant groupings	We did not constrain our sample to any particular social group. Participants were recruited from UC Berkeley's Research Participation Program which provides extra course credit for students taking Psychology courses. We collected self-report measure for the following demographic variables: Race/ Ethnicity (White / Caucasian, Hispanic / Latino, Black / African American, Native American / American Indian, Asian / Pacific Islander, Other) and education (No Schooling completed, Nursery school to 8th grade, Some high school - no diploma, High school graduate diploma or the equivalent, Some college credit - no degree, Trade/technical/vocational training, Associate Degree, Bachelor's degree, Master's degree, Professional degree, Doctorate degree).
Population characteristics	Participants for experiment 1, 2, and 3 were recruited through convenience sampling at our university. The sample consisted of healthy individuals between the ages of 18 and 40.
Recruitment	Participants in this study were recruited at UC Berkeley, specifically students participating for extra course credit in a Psychology course.
Ethics oversight	Informed consent was obtained from all participants and the study was approved by the UC Berkeley Institutional Review Board. All methods were performed in accordance with relevant guidelines and regulations of the UC Berkeley Institutional Review Board. Participants recieved course credit, 1 course credit for every hour completed in the experiment (total of 3 course credits for 3 hours).

Note that full information on the approval of the study protocol must also be provided in the manuscript.

Field-specific reporting

Please select the one below that is the best fit for your research. If you are not sure, read the appropriate sections before making your selection.

☐ Life sciences ☒ Behavioural & social sciences ☐ Ecological, evolutionary & environmental sciences

For a reference copy of the document with all sections, see nature.com/documents/nr-reporting-summary-flat.pdf

Behavioural & social sciences study design

All studies must disclose on these points even when the disclosure is negative.

Study description	All experiments in this study were behavioral studies which involved continuous ratings on a 2-dimensional grid. Observers were assigned to either the context-only, character-only, or ground-truth condition randomly (Experiments 1 & 2) or completed all three conditions (Experiment 3). All data collected in this study is quantitative.
Research sample	In Experiment 1, there was a total of 593 participants (397 females, 193 males, 3 other) ranging in age from 18 to 33 (M = 22.5, SD = 1.95 years). Part of the data from participants in Experiment 1 was retrieved from Ortega, Chen, & Whitney (2023).<br><br>In Experiment 2, data was retrieved from Chen & Whitney (2019) and had a total of 227 participants.<br><br>In Experiment 3, there was a total of 124 participants (87 females, 34 males, 2 Non-binary/non-conforming, 1 Prefer not to say) ranging in age from 18 to 40 (M = 21.46, SD = 3.42 years).
Sampling strategy	Participants for all experiments were recruited through convenience sampling at our university. Sample size for Experiment 1 and 2 were predetermined by Ortega, Chen, & Whitney (2023) and Chen & Whitney (2019) and were not pre-considered for this study. The sample size for Experiment 3 in this study was decided based on a power analysis calculated using G*Power software to detect a small-medium effect size (r = 0.25) between matched pairs. Results indicated that 101 subjects would be needed to reach a 1 - β = .8. We oversampled for an additional 20% to account for potential attrition based on online data collection or inattentive participants, leading to a total sample size of 124.<br><br>No potential self selection bias was expected for this study.
Data collection	Participants completed the experiment by themselves either in-lab or online using their personal computer. The experiment was run on PsychoPy (v2022.1.1). Researcher assistants running the study (in-lab portion) were blind to the experimental condition and study hypothesis.
Timing	Data collection for Experiment 3 ran from January 2022 to May 2022.

Data exclusions	Ten participants from Experiment 3 were removed from the analysis due to data collection issues resulting from the online website crashing during the experiment before data was saved.
Non-participation	No participants dropped out or declined participation in our study.
Randomization	In Experiment 1 and 2, observers were randomly assigned to one of the three rating conditions (ground truth, character-only, and context-only). In Experiment 3, all participants completed all three conditions, however the order that they completed each condition as counterbalanced across participants.

Reporting for specific materials, systems and methods

We require information from authors about some types of materials, experimental systems and methods used in many studies. Here, indicate whether each material, system or method listed is relevant to your study. If you are not sure if a list item applies to your research, read the appropriate section before selecting a response.

Materials & experimental systems

n/a	Involved in the study
☒	☐ Antibodies
☒	☐ Eukaryotic cell lines
☒	☐ Palaeontology and archaeology
☒	☐ Animals and other organisms
☒	☐ Clinical data
☒	☐ Dual use research of concern
☒	☐ Plants

Methods

n/a	Involved in the study
☒	☐ ChIP-seq
☒	☐ Flow cytometry
☒	☐ MRI-based neuroimaging

Plants

Seed stocks	N/A
Novel plant genotypes	N/A
Authentication	N/A
